# Telomere Maintenance Associated Mutations in the Genetic Landscape of Gynecological Mucosal Melanoma

**DOI:** 10.3389/fonc.2020.01707

**Published:** 2020-09-02

**Authors:** Guangwen Yuan, Jinge Song, Ning Li, Qianqian Song, Yifei Li, Yingxi Du, Xiaobing Wang, Yuchen Jiao, Lingying Wu

**Affiliations:** ^1^Department of Gynecological Oncology, National Cancer Center/National Clinical Research Center for Cancer/Cancer Hospital, Chinese Academy of Medical Sciences and Peking Union Medical College, Beijing, China; ^2^State Key Laboratory of Molecular Oncology, National Cancer Center/National Clinical Research Center for Cancer/Cancer Hospital, Chinese Academy of Medical Sciences and Peking Union Medical College, Beijing, China; ^3^Department of Blood Transfusion, Peking University Third Hospital, Beijing, China

**Keywords:** *ATRX*, alternative lengthening of telomeres, gynecological melanoma, *TERT*, telomere maintenance mechanisms

## Abstract

**Purpose:**

Gynecological melanomas (GMs) are rare tumors with a poor prognosis. Here, we performed exome sequencing to generate the mutational landscape of GMs.

**Methods:**

Next-generation sequencing was carried out on mucosal melanoma samples (*n* = 35) obtained from gynecological sites. The alternative telomere lengthening (ALT) phenotype was verified by fluorescence *in situ* hybridization and the C-circle assay. Immunohistochemistry was performed to detect ATRX protein. Copy number variations in *TERT* were detected by droplet digital polymerase chain reaction.

**Results:**

In the 58 formalin-fixed paraffin-embedded samples, we identified 33 (56.9%) ALT-positive cases, with 23 showing loss of ATRX protein. *TERT* promoter mutation was not detected in GMs (*n* = 40), but copy number variations in the *TERT* region were observed in 20% (7/35) of the samples. *TERT* amplification was mutually exclusive with ALT (*P* < 0.05). Kaplan–Meier revealed that ALT relative to *TERT* amplification was associated with longer overall survival in GM patients without metastasis.

**Conclusion:**

These findings indicate that telomere maintenance mechanisms play a critical role in the tumorigenesis of GMs and may aid in the prediction of clinical prognosis and the development of targeted therapy for the treatment of GM.

## Introduction

Melanoma causes about 10,000 deaths in the United States every year and thousands more worldwide (1). In China, approximately 8,000 new cases and 3,200 deaths are estimated to have occurred in 2015 (2). However, the epidemiology is strikingly different between Caucasians and Asians. Cutaneous melanoma (CM) caused by ultraviolet radiation is the major subtype in Caucasians, whereas acral lentiginous melanomas (ALMs) and mucosal melanomas occur at a higher frequency in Asians (3, 4). Mucosal melanoma is characterized by occurrence on sun-shielded skin sites such as head and neck, the anorectum, and the female genital tract. The incidence of mucosal melanoma is higher among females than males, which is due to the relatively high incidence of genital tract melanomas occurring predominantly in the vulva and vagina (4).

Although the molecular characterization of ALMs and mucosal melanoma remains limited, some notable differences from CMs have been identified. For example, *BRAF* mutation is one of the most commonly mutated genes in CMs (40–60%) (5, 6), but appears in only approximately 25% of ALMs and mucosal melanomas (7). The molecular mechanisms driving mucosal melanoma development may be distinct and thus lead to specific cancer treatments.

Cancer development involves the progressive corruption of many antitumor molecular mechanisms, some of which bypass the critical checkpoint control of telomere shortening, which enable indefinite proliferation. Telomere maintenance mechanisms (TMMs) include telomerase activation or a telomerase-independent pathway called alternative lengthening of telomeres (ALT) (8). ALT cells show a set of unusual characteristics, including the generation of extrachromosomal telomere repeats, the presence of ALT-associated PML bodies, and frequent intrachromosomal and interchromosomal telomere exchange (9), which is associated with homologous recombination. ALT occurs in 10 to 15% of human cancers, including osteosarcoma, and glioblastoma (10). However, most cancer cells maintain telomere length through telomerase activation. Telomerase is a complex of a reverse transcriptase, TERT, and an RNA moiety, TERC (telomerase RNA component), which synthesizes telomeric DNA repeat sequences at the 3′ end of a linear chromosome (11, 12). Somatic mutations in various genes have been associated with these two different TMMs. For instance, in CM, the dominant variation about TMM is *TERT* promoter mutation, whereas ALT-associated mutations have never been reported (13). ALT is also associated with genetic inactivation or loss of expression of the histone H3.3, and the chaperone proteins ATRX and DAXX (10), which incorporate H3.3 (encoded by H3F3A or H3F3B) into telomeric and pericentric chromatin (14). Finally, a recent study demonstrated that mutation of SMARCAL1 could activate the ALT phenotype independent of ATRX mutation (15).

In this study, we performed exome sequencing on 35 mucosal melanoma samples obtained from gynecologic sites (GMs), which yielded a genetic landscape that was significantly different from that of CMs. The characteristic mutational pattern in the TMM-associated genes indicated distinct TMM in GMs, which were further validated with fluorescence *in situ* hybridization (FISH) or droplet digital polymerase chain reaction (ddPCR). This study highlights the significance of telomere maintenance alterations in the tumorigenesis of GMs and provides potential diagnostic markers and therapeutic targets for these melanomas.

## Materials and Methods

### Patient Specimens

The study was approved by the Institutional Review Board of Cancer Hospital, Chinese Academy of Medical Sciences (trial registration ID: 19/221-2005). Tumor samples were obtained from Chinese female patients with GMs at Cancer Hospital, Chinese Academy of Medical Sciences, with written informed consent. Matched normal samples were not available for four cases. Clinical information, including age, tumor site, pathological stage (according to TNM classification), overall survival (OS), and status, was collected (Supplementary Table S1). Sequencing data for CM (TCGA-SKCM) samples (*n* = 468) were obtained from The Cancer Genome Atlas (TCGA) and for ALMs and uveal melanomas (UVMs) from previous studies (6, 16–19).

### Study Design

At first, whole-exome sequencing (WES) was performed on samples with frozen tissues (*n* = 20). Then formalin-fixed paraffin-embedded (FFPE) samples (*n* = 58; including 20 cases with frozen tissues) were screened with FISH. After that, we sequenced the exome in ALT-positive FFPE samples with sufficient DNA. Sanger sequencing and ddPCR were applied to detect *TERT* variations in ALT-negative FFPE samples and ALT-positive FFPE samples without enough DNA to finish WES. Cases with both FFPE and frozen tissue samples (*n* = 20) participated in all experiments because sufficient DNA could be extracted. Finally, immunohistochemistry (IHC) was conducted on FFPE samples for ATRX protein detection. The schematic diagram and the detailed information for each sample are indicated in Supplementary Figure S1 and Supplementary Table S1.

### DNA Extraction

DNA was extracted from frozen tumor tissues or white blood cells (WBCs) using the QIAamp DNA Mini Kit (51306; Qiagen; Germantown, MD, United States). FFPE DNA was extracted using the Gene Read FFPE DNA Kit (1080391; Qiagen). All samples were quantified using the Qubit dsDNA HS Assay (Q32866; Life Technologies/Thermo Fisher Scientific, Waltham, MA, United States).

### WES and Data Analysis

Whole-exome sequencing was performed on DNAs isolated from fresh-frozen GM tissues (*n* = 20) and ALT-positive FFPE samples (*n* = 15) selected through FISH analysis of 58 FFPE samples. The normal control for three samples was not available, and FFPE DNAs for another six ALT-positive samples could not be isolated because of insufficient tissue (Supplementary Figure S1).

DNA library preparation for tumor-normal pairs (*n* = 32) and tumor-only samples (*n* = 3; FFPE samples with insufficient normal tissue) was performed using the KAPA HyperPrep Kit (KK8504; KAPA Biosystems; Wilmington, MA, United States) and captured using next-generation sequencing exome probes (V5-5990-9857EN; Agilent, Santa Clara, CA, United States) with the SureSelect QXT Target Enrichment Kit-ILM-Hyb Module Box2 (5190-7334, Agilent). All captured libraries were sequenced on an Illumina HiSeq2000 platform (Illumina, San Diego, CA, United States). The sequencing data were shared on Genome Sequence Archive (ID: CRA002180; CRA002926).

We identified somatic variants using the GATK Best Practices Pipeline (software.broadinstitute.org; Broad Institute, Boston, MA, United States). After Illumina sequencing, all produced FASTQ reads were quality-checked and trimmed with FastQC (version 0.11.2; http://www.bioinformatics.babraham.ac.uk/projects/fastqc/) and Trimmomatic (version 0.33) (20). Sequencing reads were aligned to human genome hg19 with the BWA MEM software for both tumor and normal samples (21). Picard software (version 1.103; https://broadinstitute.github.io/picard/) was used to mark PCR duplications of each BAM file, which was locally realigned, and the base quality scores were recalibrated with GATK (version 3.1) (22). MuTect (version 1.1.6) was used to call single-nucleotide variants (SNVs) with default parameters (23). Only SNVs that were classified as “KEEP” by MuTect were used for downstream analysis. Indels were called using Strelka (version 1.0.14) with default parameters (24). All the somatic variants were validated with IGV and annotated with Variant Effect Predictor (version 83; Supplementary Tables S2–S4) (25). We analyzed mutation signature of 32 samples using the Wellcome Trust Sanger Institute mutational signatures framework with the algorithm known as non-negative matrix factorization (26).

### ALT FISH

Telomere length was assessed using FISH in the 58 samples with available FFPE tissues. Briefly, deparaffinized slides were hydrated, steamed for 20 min in citrate buffer (H-3300; Vector Laboratories, Burlingame, CA, United States), dehydrated, and hybridized with a Cy3-labeled peptide nucleic acid (PNA) probe complementary to the mammalian telomere repeat sequence [(N-terminus to C-terminus; CCCTAA)_3_]. Posthybridization washes were performed, followed by nuclear counterstaining with DAPI. Fluorescence images were captured under fluorescence microscopy (Leica DM2500, Buffalo Grove, IL, United States) using CytoVision (Leica).

Fluorescence *in situ* hybridization slides were assessed and scored independently. Large, ultrabright telomere repeat DNA aggregates are unique to ALT-positive cell populations and are larger and brighter than FISH signals emanating from individual telomeres in the same samples (detailed discussion of these foci as surrogate markers for the ALT phenotype can be seen below).

Alternative telomere lengthening-positive and -negative GMs were classified using the following criteria: ALT-positive samples (i) neoplastic cells demonstrated individual telomeric foci with the presence of intranuclear foci, ultrabright telomere FISH signals; and (ii) >1% of tumor cells displayed these alternative lengthening of telomeres-associated telomeric foci; ALT-negative samples: tumor samples in which no ALT-associated telomeric foci were found in at least 5,000 cells were considered ALT-negative. Areas showing necrosis were not included in the evaluation. The specific criteria used for interpreting the FISH result were the same as those defined previously (27).

### C-Circle Assay

We performed rolling circle amplification of C-circles as described (28). For each sample, the assay was performed with and without phi29 polymerase (M0269S; New England Biolabs, Ipswich, MA, United States). Seven microliters of each sample (10 ng) was mixed with 3 μL of 10 μg/μL bovine serum albumin, 10% Tween, 10% DTT, dNTP mix, Phi29 buffer, and Phi29 DNA polymerase and incubated at 30°C for 8 h followed by 65°C for 20 min.

C-circle assay products were diluted 1:4 in Tris–EDTA (10 mM Tris, 0.1 mM EDTA, pH 7.6), and 5 μL of the diluted product was used for each PCR (triplicate telomere PCR of CC assay/phi29+, triplicate telomere PCR of C-circle assay/phi29-). The qPCR master mix contained the following: 1 × QuantiTECT SYBR Green Master Mix (4309155; Life Technologies/Thermo Fisher Scientific), 0.1 M DTT, 0.5 μL dimethyl sulfoxide, and 10 μM of both forward and reverse primers. PCR conditions were the following: 95°C for 15 min, 35 cycles of 95°C for 15 s, and 54°C for 2 min. The C-circle signal for a telomerase positive cancer cell line, HeLa (negative control), as well as for a prototype of ALT-positive cells, U2OS (positive control), were measured under identical conditions. The cell lines were acquired from ATCC.

### ATRX IHC

A subset of tumors (*n* = 47) was analyzed for ATRX nuclear staining. Antigen retrieval was performed in a steamer using citrate buffer (H-3300; Vector Laboratories) for 30 min. Endogenous peroxidase was blocked (PV9001; ZSGB-BIO, Beijing, China), and serial sections were then incubated with primary antibody against ATRX (1:100 dilution; sc-55584; Santa Cruz, Dallas, TX, United States) overnight at 4°C. To detect primary antibody, slides were incubated with secondary antibody (PV9001, ZSGB-BIO) for 30 min, counterstained with Harris hematoxylin, rehydrated, and mounted. Staining of the GM slides was assessed and scored; epithelial cells and stromal cells were used as internal controls to display nuclear staining for ATRX (Supplementary Figure S2). ATRX expression in all samples is listed in Supplementary Table S5.

### *TERT* Promoter Sequencing and *TERT* Gene Copy Number Determination

Targeted amplification and sequencing were performed on the *TERT* promoter region containing the two most common mutations identified in human cancers, C228T, and C250T. The following primers were used for PCR amplification (489bp) and Sanger sequencing of DNAs isolated from fresh-frozen tissue samples: *hTERT*-F 5′-gtaaaacgacggccagt-GGCCGATTCG ACCTCTCT-3′ and *hTERT*-R: 5′-AGCACCTCGCGGTAGTGG-3′. The sequences of the primers used for amplification of DNA from FFPE samples were the following: *hTERT*-FFPE-F: 5′-ccaggccgggctcccagt-3′ and *hTERT*-FFPE-R: 5′-gaaggggagg ggctgggagg-3′. PCR was performed in a total reaction volume of 50 μL, containing 25 μL 2 × GoldStar MasterMix (CW0939S; CWBIO, Beijing, China), 2 μL primer F (10 mM), 2 μL primer R (10 mM), 30 ng DNA template, and ddH_2_O to 50 μL. The following cycling conditions were used: 95°C for 10 min, followed by 35 cycles of 95°C for 30 s, annealing at 65°C for 30 s, and polymerization at 72°C for 1 min.

*TERT* gene copy number variants were quantified with the QuantStudio^TM^ 3D Analysis Suite^TM^ according to the manufacturer’s instructions (Thermo Fisher Scientific). Chips were read in the Quantstudio 3D chip reader to obtain raw fluorescence values and analyzed on QuantStudio^TM^ 3D AnalysisSuite^TM^. ddPCR reagents are partitioned into 20,000 droplets before reactions proceed to the reaction plateau end point in individual droplets. Droplets are evaluated as positive or negative based on fluorescence signal intensity. A representative quantitative map is in Supplementary Figure S3. For each sample, a specified amount of DNA template was used as the expected quantity (ng). After the template was combined with the Taqman probe (Applied Biosystems^TM^ TaqMan^TM^ Liquid Biopsy dPCR Assay, A44177; Applied Biosystems, Waltham, MA, United States) and amplified with the primers, the actual copy numbers were read and converted into the measured quantity (ng). The ratio of the measured quantity A (ng) to the expected quantity B (ng) times the number of reference species copies N_B_ in the human genome (copy number = A/B × N_B_) was used to calculate copy number. The copy number of the *TERT* promoter in normal cells was 2 copies/cell as detected in WBCs (Supplementary Table S6). The threshold for *TERT* amplifications was 4 copies/cell and for loss, 1 copy/cell.

### Statistics

Statistical significance was determined with one-way analysis of variance (ANOVA; Sidak multiple-comparisons test) and the Fisher exact test. The log-rank (Mantel–Cox) test was used in Kaplan–Meier analysis. For determining presence of C-circles with qPCR, a Δmean Ct value (Δmean Ct = mean Ct of non-Φ29–treated samples −Φ29-treated sample) was calculated for each sample. A Δmean Ct value > 0.5 was considered positive (Supplementary Table S7), and results were also confirmed using the dot blot. Four levels of statistical significance were considered: *P ≤ 0.05; **P ≤ 0.01; ***P ≤ 0.001, and ****P ≤ 0.0001.

## Results

### The Genomic Landscape of Gynecological Melanoma

**FIGURE 1:**
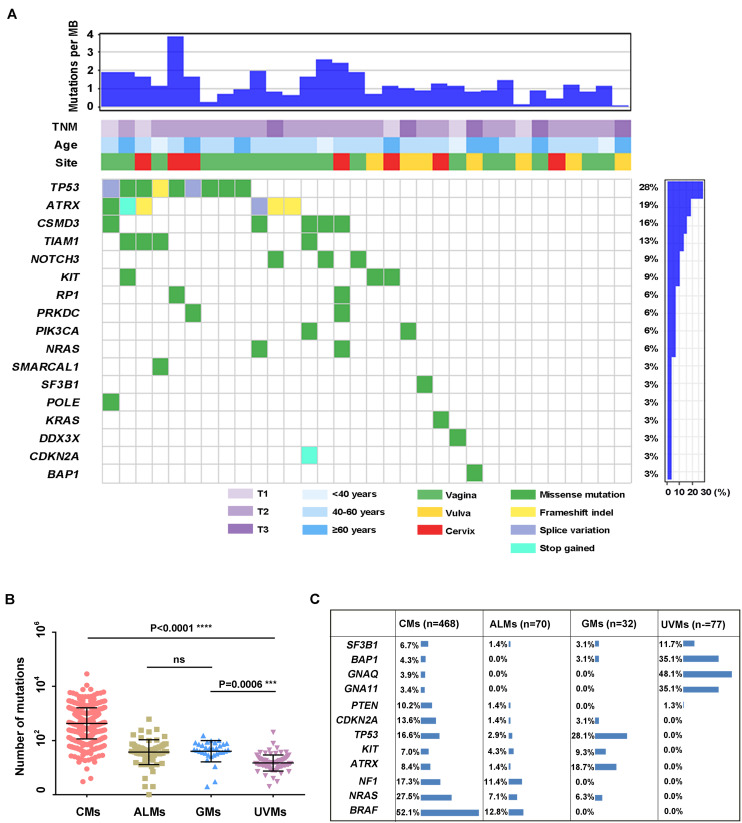
Summary of mutations detected in DNAs isolated from primary GM samples (*n* = 32). **(A)** Mutations and clinical data compiled for tumors from individual patients. Shown are the total number of somatic mutations in coding regions, mutations in frequently mutated genes in each sample, age at diagnosis, primary site, and TNM stage; **(B)** mutational load in different melanoma subtypes: CMs (*n* = 468), ALMs (*n* = 70), GMs (*n* = 32), and UVMs (*n* = 77; one-way ANOVA; Sidak multiple-comparisons test; 95% confidence interval); and **(C)** the frequency of selected published melanoma driver genes in CMs (the mutation frequency in TCGA database includes synonymous mutations), ALMs, GMs, and UVMs.

To investigate the genetic landscape of GM, we performed exome sequencing on 32 pairs of matched tumor/normal samples and three tumor samples without matched normal tissue. In the 32 pairs of samples, 1,646 somatic SNVs and 91 somatic indels were identified. The median number of somatic mutations in coding regions (missense, splice site, inframe indels, frameshift, start lost, and stop lost/gain) was 49 (range of ∼2 to 137 SNVs; Figure 1A and Supplementary Table S3). We compared the tumor mutational load of GMs with that of CMs, UVMs, and ALMs from the TCGA or previous studies (*Methods*; Figure 1B). The tumor mutational load was significantly higher in CMs (*n* = 468) than in the other three subtypes (*P* < 0.0001; one-way ANOVA; Sidak multiple-comparisons test; and 95% confidence interval). GMs (*n* = 32) and ALMs (*n* = 70) sustained more mutations than UVMs (*n* = 77; GMs vs. UVMs, *P* = 0.0001; ALMs vs. UVMs, *P* = 0.0006). There was no significant difference in the tumor mutational load between GMs and ALMs. Oncogenic mutations in *BRAF* were absent in GMs, although it was the most frequently mutated gene in CMs. Other commonly mutated genes in CM and UVM, such as *NRAS*, *GNA11*, and *GNAQ*, were also rarely altered in GMs (Figure 1C). Thus, the mutational landscape of GMs was distinct from the other subtypes of melanoma (Figure 1C).

**FIGURE 2:**
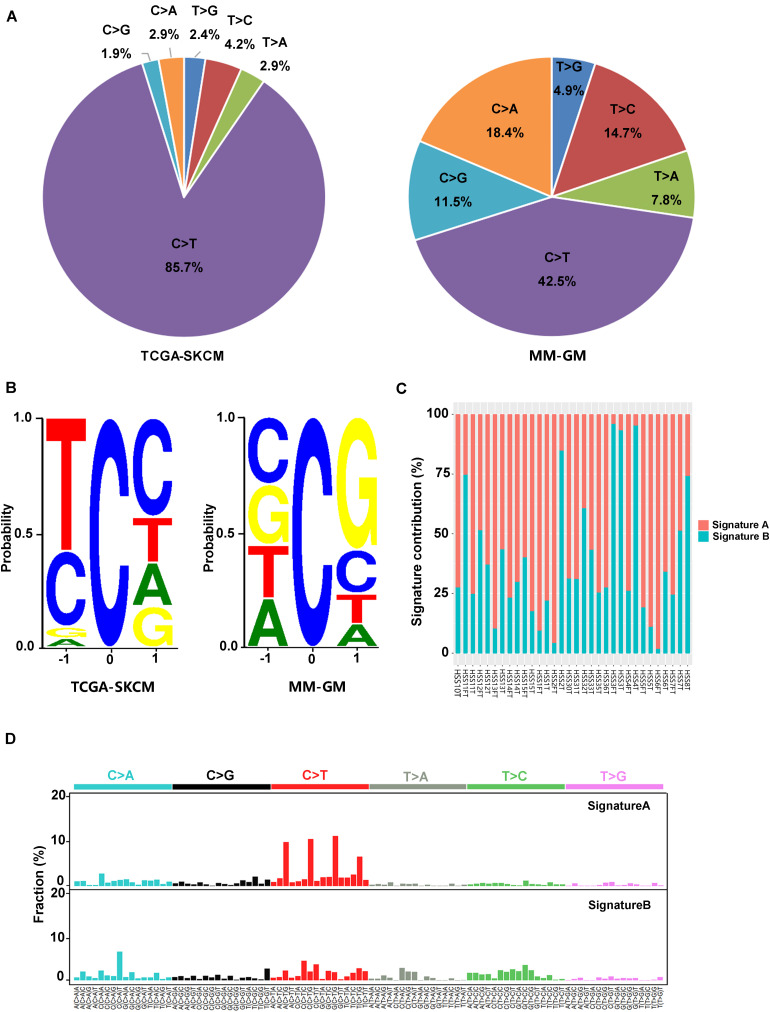
Mutation signatures in 32 GMs are distinct from CMs. **(A)** Percentage of the six possible mutation classes in the exomes of TCGA-SKCM (*n* = 468) and MM-GM; **(B)** sequence contexts of C > T mutations in TCGA-SKCM (*n* = 468) and MM-GM. The height of each base indicates the probability that the base appears at the position flanking the mutated cytosine; **(C)** distribution of the two signatures in each patient; and **(D)** pattern of two signatures (signatures **A,B**) observed in 32 GMs. The mutation types are displayed on the horizontal axes, and the percentage of mutations attributed to a specific mutation type on the vertical axes. MM-GM, gynecological mucosal melanoma.

In the 32 GM cases, the percentage of C > T (equal to G > A) transitions reached 42.5% (Figure 2A). Other transition types with significant frequency in GMs included the following: C > A (equal to G > T) at 18.4%, T > C at 14.7%, and C > G at 11.5% (Figure 2A). These features were significantly different from the mutational signature of CMs associated with ultraviolet light exposure, in which C > T conversions appeared at a frequency of 85.7% (Figure 2A). In addition, there was a preference for a G in the position following the mutated C residue in GMs. However, CMs exhibited a strong preference for C in the position before and after the mutated C for C > T mutations because the ultraviolet-related signature is associated with frequent CC > TT dinucleotide mutations at dipyrimidines (Figure 2B). Two signatures (signature A and signature B) in GMs were identified with non-negative matrix factorization (26) based on the mutations identified from the exome sequencing (Figures 2C,D). We compared these two signatures to the Signatures of Mutational Processes in Human Cancer in the COSMIC database (29) and intended to figure out whether the known signatures have implication for the pathogenesis and mutational process of GMs. Signature A (Figure 2D), which predominated in 23 GM patients (proportion of signature A > 50% in each patient), showed a strong correlation [0.9306; cosine similarity value (CSV)] to the signature 1 from the COSMIC database. Signature 1, which is generated through endogenous spontaneous deamination of 5-methylcytosine and correlated with age cancer diagnosis, has been found in all cancer types. Signature B (Figure 2D) showed some similarity to both signature 5 (CSV = 0.716), which is ubiquitous in many types of cancers of unknown etiology, and signature 3 (CSV = 0.6756), which represents failure of DNA double-strand break-repair by homologous recombination. Signature 3 is also associated with germline and somatic *BRCA1* and *BRCA2* mutations in breast, pancreatic, and ovarian cancers. However, signature B did not correlate with any other known signatures in the COSMIC database with a dominant similarity. Signature 7, an important signature in CMs potentially caused by ultraviolet exposure, was not found in GMs.

**FIGURE 3:**
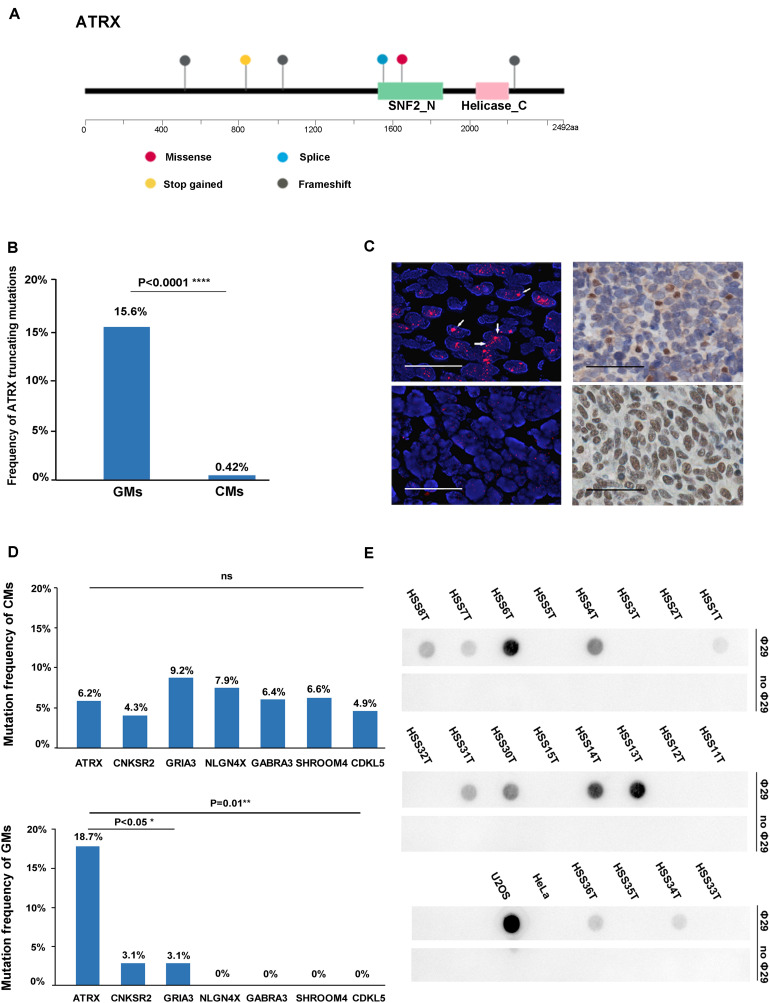
ATRX variations in the gene, protein, and function. **(A)** Diagram of the location of mutations across the coding region of *ATRX* in six GMs; **(B)** graphic representation of the frequency of truncating mutations in GMs (*n* = 32) and CMs (*n* = 468); **(C)** mutation frequency of similar-sized large genes on chromosome X: *CNKSR2* (20/468,4.3%; chrX:21,393,016-21,659,655; and size: 266,640 bp), *GRIA3* (43/468,9.2% chrX:122,318,388-122,616,895; and size: 298,508), *NLGN4X* (37/468,7.9%; chrX:5,808,083-6,146,706; and size: 338,624 bp), *GABRA3* (30/468, 6.4%; chrX:151,335,634-151,619,831; and size: 284,198 bp), *SHROOM4* (31/468, 6.6%; chrX:50,334,643-50,557,044; and 222,402 bp), and *CDKL5* (20/468, 4.3%; chrX:18,443,725-18,671,749; and size: 228,025 bp) compared to *ATRX* in CMs and GMs, respectively; **(D)** Left: representative images of FISH performed with a PNA probe to detect telomeres. (Top) Example of an ALT-positive tumor with an *ATRX* mutation (HSS8T). Large, ultrabright telomere FISH signals (red) indicative of ALT are highlighted by the arrows. (Bottom) Example of an ALT-negative GM without an *ATRX* mutation (HSS2T; scale bars, 0.1 mm); right: representative images of immunohistochemistry performed to detect *ATRX* in the same tumors. (Top right) HSS8T harboring *ATRX* mutation, and in the (bottom right), HSS2T harboring wild-type *ATRX* (scale bars, 0.25 mm); **(E)** C-circle dot blot. Rows marked “Φ29” were treated with Φ29 polymerase to amplify C-circles. Prominent dots in these rows represent the presence of C-circles. Rows marked “without Φ29” as internal controls represent the group without amplification.

Several recurrently mutated genes were identified in GMs. *TP53* was the most frequently mutated gene in GMs (28.1%), which had no significant difference compared with CM (16.6%; Fisher exact test, *P* = 0.143). *ATRX* was the second frequently mutated gene (6/32, 18.7%), followed by *CSMD3* (5/32, 15.62%), *TIAM1* (4/32, 12.5%), *NOTCH3* (3/32, 9.4%), and *KIT* (3/32, 9.4%; Figure 1A). Among these genes, the frequency of *ATRX* mutations exhibited the most significant difference between GMs and CMs. The frequency of *ATRX* mutations in GMs (6/32, 18.7%) was more than three times that of *ATRX* mutations in CM cases (29/468, 6.19%; Fisher exact test, *P* = 0.018; the mutation frequency in TCGA database excluded synonymous mutations). Furthermore, five of the six *ATRX* mutations in GMs were truncating mutations (three frameshift, one stop gained, and one splice site; Figure 3A). In CMs, most *ATRX* mutations were missense mutations (27/29). The frequency of *ATRX* truncating mutations was only 0.42% (2/468) in CMs, which was significantly different from the frequency in GMs [15.6% (5/32); *P* < 0.0001; Fisher exact test; Figure 3B]. To test whether the missense mutations were random in CMs, we selected six genes on chromosome X similar size to the coding region with *ATRX*. The mutation frequency of these six genes was similar to *ATRX* in CMs, but significantly lower than the mutation frequency of *ATRX* in GMs (Figure 3C). These results indicated that most of the *ATRX* mutations in CMs tended to be passenger mutations as a consequence of the large size of the coding region and the high mutational burden.

In addition, three of six *ATRX*-mutated GMs harbored *TP53* mutations. We also detected an *ATRX* truncating mutation accompanied by *TP53* mutation in a GM case without matched normal tissue (Supplementary Table S4).

### Gynecological Melanomas Are ALT Positive

To demonstrate the potential oncogenic function of the mutations in the telomere maintenance pathways in GMs, we performed FISH with a PNA probe to assess telomere status in 58 cases. Thirty-three of the 58 GMs (56.9%) were classified as ALT-positive, and the remaining 25 (43.1%) as ALT-negative (Figure 3D and Supplementary Table S5). In order to establish the accuracy of the FISH to detect ALT, we compared these results with the C-circle assay. Twenty samples were analyzed for the presence of ALT-specific C-circles. Based on the results of the dot blots (Figure 3E) and qPCR (Supplementary Table S7; the outcomes of the dot blots and qPCR showed no difference), the samples that were positive in the C-circle assay were also positive in FISH. For only one sample, HSS3T, there was a discrepancy between the two assays. HSS3T was positive for ALT based on FISH but negative in the C-circle assay. The proportion of ALT-positive tumor cells was lower in HSS3T, and hybridization signals were indeed not as strong relative to the other samples positive by FISH (Supplementary Figure S4). When DNA is extracted from tumor cells, as for the C-circle assay, positive signals will be weakened, resulting in the absence of hybridization on dot blots. However, FISH, which is the gold standard for the detection of ALT, detects changes in telomeres in single cells. Therefore, we considered HSS3T to be ALT-positive. Among the 23 ALT-positive cases with exome sequencing data in paired tumor/normal samples, *ATRX* mutations were found in six samples, and a *SMARCAL1* mutation was identified in one case. *H3F3A* and *DAXX* mutations were not found in these samples.

We performed IHC to assess the relationship between ATRX protein levels and the mutation status of *ATRX* in the 47 GMs. All six samples with *ATRX* mutations showed loss of nuclear ATRX expression. Altogether, 23 of 28 (82.1%) ALT-positive samples showed loss of nuclear ATRX staining in tumor cells (Figure 3D). Immunostaining was positive for ATRX in 5 (17.9%) ALT-positive samples, but ATRX mutations were not present based on the exome sequencing data. One of these cases did harbor a *SMARCAL1* mutation. All 19 ALT-negative samples showed positive nuclear staining for ATRX protein (Table 1 and Supplementary Table S5). In summary, these results demonstrated that the loss of ATRX expression was closely related to the ALT phenotype in GMs (*P* < 0.001, Fisher exact test).

**TABLE 1:** Relationship of ALT pathway with *ATRX* expression and *TERT* amplification in GMs.

	*ATRX* expr.			*TERT* amp.		
			
	+	−		+	−	
			
	*n* (%)	*n* (%)	*P*	*n* (%)	*n* (%)	*P*
FISH			<0.001			<0.05
ALT negative	19/47 (40.4%)	0/47 (0%)		7/35 (20%)	13/35 (37.1%)	
ALT positive	5/47 (10.6%)	23/47 (48.9%)		0/35 (0%)	15/35 (42.9%)	

*FISH, fluorescence *in situ* hybridization; ALT, alternative telomere lengthening.*

### *TERT* Alterations in GMs

**FIGURE 4:**
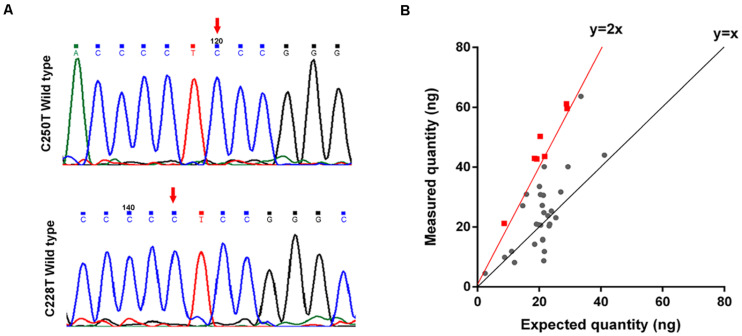
*TERT* is amplified in some GMs. **(A)** Chromatogram from Sanger targeted sequencing performed on the *TERT* promoter at the commonly mutated nucleotides C228T and C250T. No mutation was found in 40 tumors; **(B)** ddPCR to detect *TERT* copy number variations (*n* = 35). The measured quantity of DNA (ng) is plotted against the expected quantity of DNA (ng) where the control is normal DNA. Samples with at least 4 copies/cell (the red dot on the red line or on the left side) were considered to have *TERT* amplification (*n* = 7).

To determine the mechanisms underlying TERT activating in the tumorigenesis of GMs, we performed Sanger sequencing of the *TERT* promoter region on 40 samples. No mutations, including C228T and C250T, were identified in the *TERT* promoter region of these 40 samples (Figure 4A). This result for GMs was significantly different from CMs where *TERT* promoter mutations were found in greater than 50% of samples (13).

We next investigated whether other genetic alterations were present that could lead to the activation of telomerase. We examined *TERT* gene copy number variations in 35 GMs using ddPCR. Amplification of the *TERT* region was detected in 20% (7/35) of the samples (copy number > 4 copies/cell; Figure 4B and Supplementary Table S6), and all were ALT-negative cases (Table 1). In these cases, *TERT* amplifications, rather than *TERT* promoter mutations, might contribute to the tumorigenesis of GMs by maintaining telomeres. Alterations in the two telomere maintenance pathways were mutually exclusive (*P* < 0.05, Fisher exact test; Table 1).

### Prognostic Value of Telomere Maintenance Mutations

**FIGURE 5:**
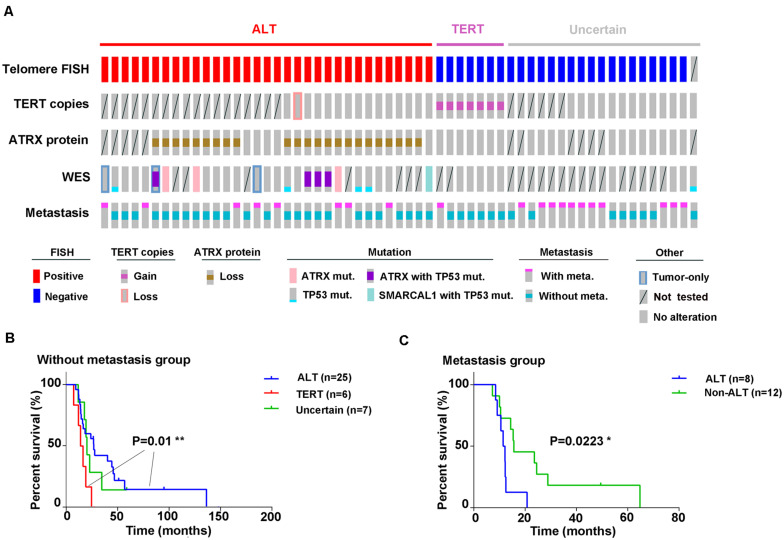
Graphical representation of all experiments performed. **(A)** Outcomes of different experiments and clinical information (disease progression) in all 59 samples are displayed graphically; **(B)** Kaplan–Meier analysis for overall survival based on ALT activity or *TERT* amplification in GM patients without metastasis [*P* = 0.01, log-rank (Mantel–Cox) test; 95% confidence interval] and in **(C)** with metastases [*P* = 0.0223, log-rank (Mantel–Cox) test; 95% confidence interval].

To determine the clinical value of the two telomere maintenance pathways in GM patients, we performed Kaplan–Meier analysis on survival data of patients based on TERT status (ALT-positive, *TERT* amplification, or uncertain; Figure 5A). In early-stage GM (stages I and II, no metastasis), the OS of patients with ALT-positive tumors showed significantly longer OS than tumors in which *TERT* was amplified [*P* = 0.01; log-rank (Mantel–Cox) test; 95% confidence interval, Figure 5B]. However, in cases of metastasis, the OS of patients with ALT-mutated tumors was shorter than in patients with non-ALT tumors [*P* = 0.0223; log-rank (Mantel–Cox) test; 95% confidence interval, Figure 5C].

## Discussion

This study represents the largest genome-wide analysis of GMs to date and the first to explore TMMs in this subtype of melanoma. The genetic landscape of GMs is highly distinct compared with CMs and possibly other mucosal melanomas. First, while two mutational signatures in GMs correlated with signature 1 and signature 5 in the COSMIC database are also the most common in acral lentiginous (*n* = 32) and mucosal melanomas (*n* = 8) (6), they are distinct from signature 7, which predominates in CMs. Second, although SF3B1 has been reported as a frequently mutated gene in mucosal melanoma (30), only one (1/32, 3.12%) SF3B1 mutation with a recurrent somatic mutation at codon R625 was found in 32 samples in this cohort. As GMs with SF3B1 mutations previously reported were primarily located in vulvovaginal sites (6, 16), the absence of SF3B1 mutations may be due to the origin of tumors in our cohort from the cervix. Finally, *TERT* promoter mutations, which are frequent in CMs (13), were absent in GM samples. Instead, we found that telomere maintenance in GMs might be mediated through other types of genetic alterations: *ATRX* loss-of-function mutations and *TERT* amplifications.

Most *ATRX* changes in GMs (83.3%; 5/6) were truncating mutations as opposed to CMs, where most were missense and occurred at a lower frequency. All the GM samples harboring *ATRX* mutations showed loss of ATRX staining. However, in a significant number of samples with loss of ATRX staining, somatic *ATRX* mutations were not detected. Because of the limitations of WES, complicated mutations in *ATRX*, such as translocations and inversions (31), were potentially missed. Epigenetic changes in *ATRX* may also lead to the loss of *ATRX* expression, while it has not been verified in our research and previous studies. In GMs, all the samples with *ATRX* mutations or loss of *ATRX* staining were ALT positive. There were five ALT-positive samples where ATRX remained intact. In one of these samples, we identified a somatic mutation in *SMARCAL1*. In a recent study, *SMARCAL1* mutations were shown to activate ALT in glioma cell lines (32). Taken together, ALT is the preferred mechanism of telomere maintenance in GMs relative to CMs, and therefore it can be considered as a potential therapeutic target specific for tumor cells. ATR kinase inhibitors promote cell death by preventing the ability of ALT-positive cells to extend telomeres through homologous recombination and promote the death of these cells (33). However, there are still no clinical trials on the therapeutic effect. Recently, Liang et al. found treatment with the AZD1775, a WEE1 inhibitor, robustly inhibited the growth of several *ATRX*-deficient cancer cell lines *in vitro*, as well as xenografts *in vivo*. AZD1775 also selectively inhibited the proliferation of patient-derived primary cell lines from gliomas with naturally occurring *ATRX* mutations (34). In our research, we found ATRX protein loss in 82.1% (23/28) of ALT-positive patients. As WEE1 inhibitors have been investigated in several phase II clinical trials, they could show efficacy in ATRX-deficient GMs.

In addition, we found a group of GMs without TMM activity (samples without ALT pathway and *TERT* amplification). Previous studies have identified a subset of CMs with no TMMs (35). An alternative to typical TMMs in these CMs was the presence of innate long telomeres, which might bypass the need to reactivate telomerase. However, we have not yet explored whether this phenomenon might be the basis for telomere maintenance in GMs, which needs to be further explored.

Finally, we examined whether ALT was associated with prognosis. The analysis revealed a complicated relationship possibly in all cancers. For example, ALT was associated with better prognosis in patients with advanced-stage pancreatic neuroendocrine tumors (PanNETs) (36). In contrast, in early-stage PanNETs, OS was shorter in patients with ALT-positive tumors (37). However, in GMs, ALT status was associated with better OS in patients without metastasis (*P* = 0.01). As the sample size was limited in this study, further analyses of more cases are needed to validate the prognostic value of ALT in GMs.

In this study, we profiled the genetic landscape of GMs. We found significant differences between GMs and CMs, the main one being activation of ALT in GMs as the TMM. The *ATRX*/ALT status could be used as a diagnostic marker and potentially a highly specific target for cancer therapy. Thus, our study provides a potential therapeutic choice for this rare type of melanoma.

## Data Availability Statement

The datasets presented in this study can be found in online repositories. The names of the repository/repositories and accession number(s) can be found below: Genome Sequence Archive (GSA; http://gsa.big.ac.cn/ID:CRA002180; CRA002926).

## Ethics Statement

The studies involving human participants were reviewed and approved by Institutional Review Board of Cancer Hospital, Chinese Academy of Medical Sciences (Trial registration ID: 19/221-2005). The patients/participants provided their written informed consent to participate in this study.

## Author Contributions

LW and YJ were the overall principal investigators who conceived the study and obtained financial support. NL, QS, YL, and YD performed sample preparation and statistical analysis and validation. JS and GY performed experimental work. JS and XW edited tables and figures. GY and JS drafted the manuscript under the supervision of LW and YJ. All authors critically reviewed the manuscript and approved the final version.

## Conflict of Interest

The authors declare that the research was conducted in the absence of any commercial or financial relationships that could be construed as a potential conflict of interest.
