# Supplementary material for: Telomere Maintenance Associated Mutations in the Genetic Landscape of Gynecological Mucosal Melanoma
Source: Front Oncol. 2020 Sep 2;10:1707. doi: 10.3389/fonc.2020.01707 (PMC7492295; doi:10.3389/fonc.2020.01707)
Supplement: Supplementary file 2 [file Data_Sheet_2.docx]

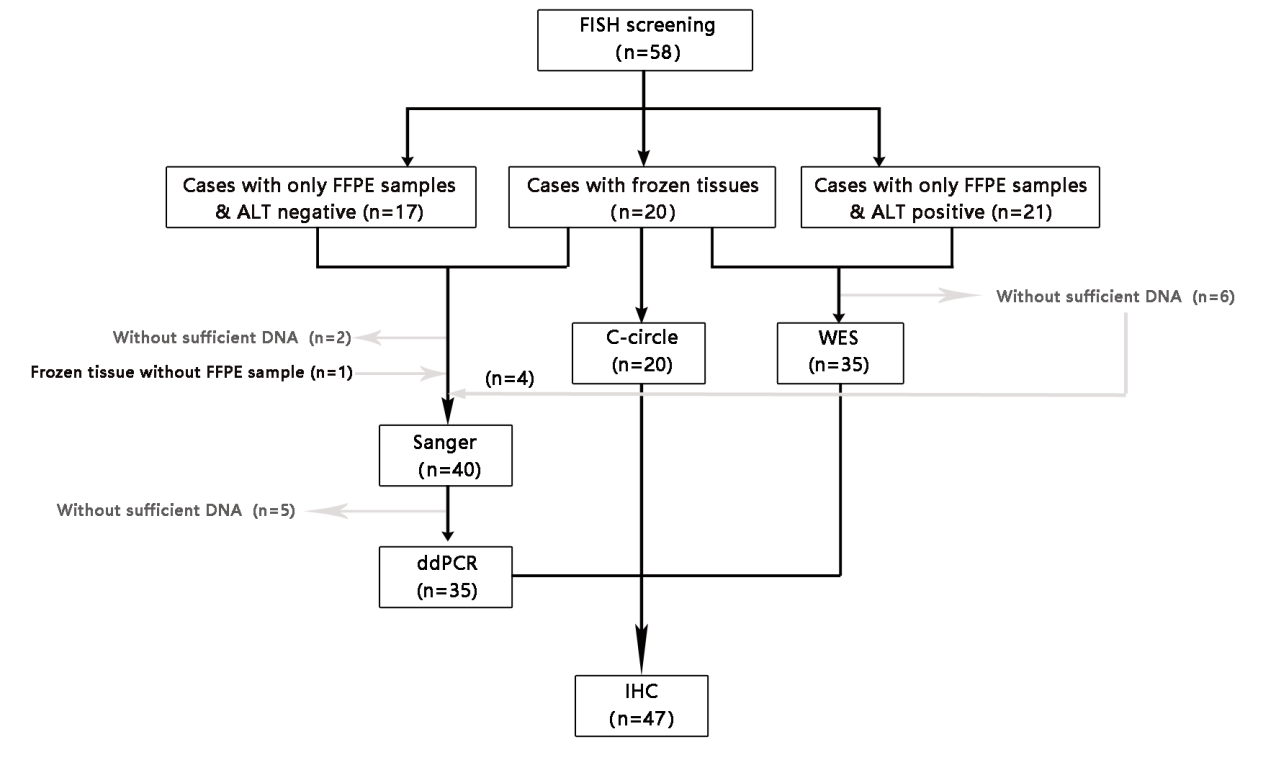


**Supplemental Figure 1. Study design.**

A flow chart to demonstrate enrollment of GM patients in each experiment.


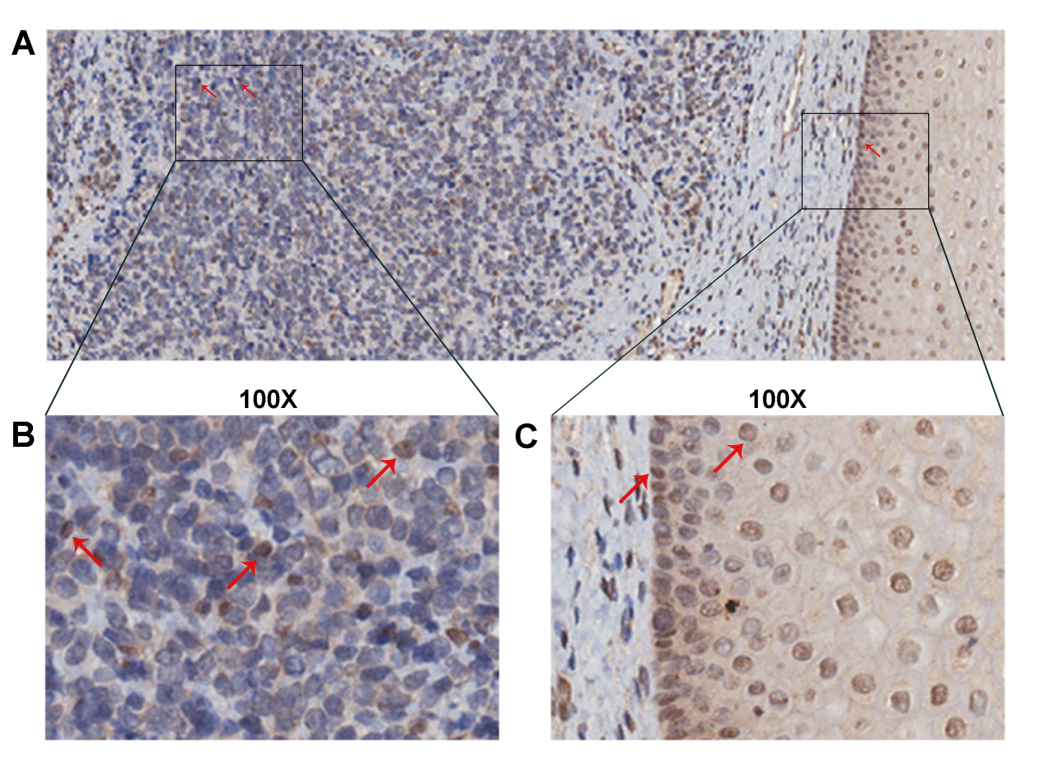


**Supplemental Figure 2. Intact and lost *ATRX* protein stained by IHC in HSS8T.**

IHC to detect ATRX protein performed on a section from case HSS8T with a truncating mutation in *ATRX*. (**A**) Coexistence of tumor and non-neoplastic cells (positive and negative for ATRX) at low magnification in HSS8T; (**B**) tumor cell nuclei (negative for ATRX) and non-neoplastic cells (positive for ATRX; marked by arrows) in tumor region; (**C**) Benign epithelial cells as a positive control for ATRX immunostaining (marked by arrows). Magnification: ×100.


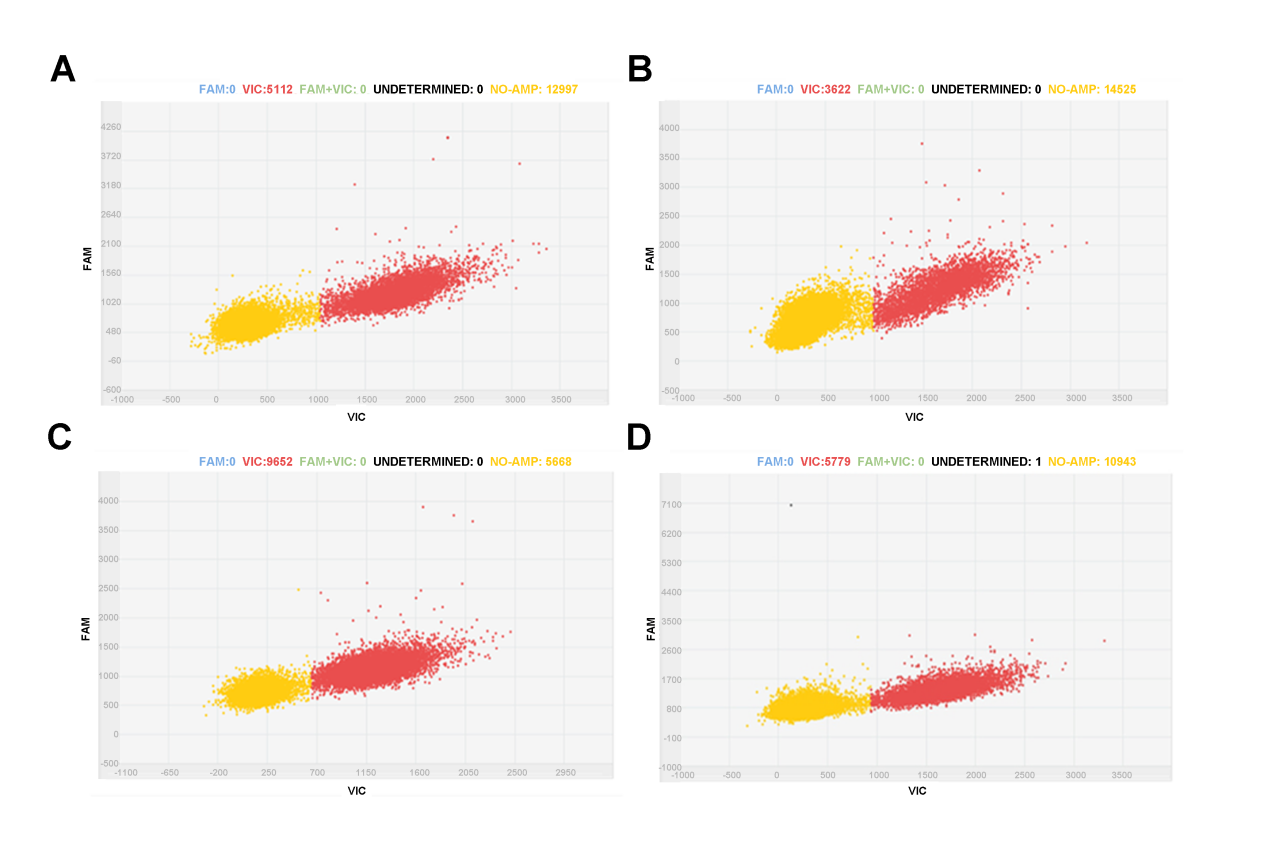


**Supplemental Figure 3. Droplet digital PCR quantitative map.**

Representative droplet digital plot for wild-type (VIC, red color) and mutant TERT promoter sequences (FAM, green color) in tumor samples. Yellow color represents droplets with no DNA. (**A**):HSS2N-TF; (**B**):HSS8N-TF; (**C**):HSS19P-TF; and (**D**): HSS20P-TF.


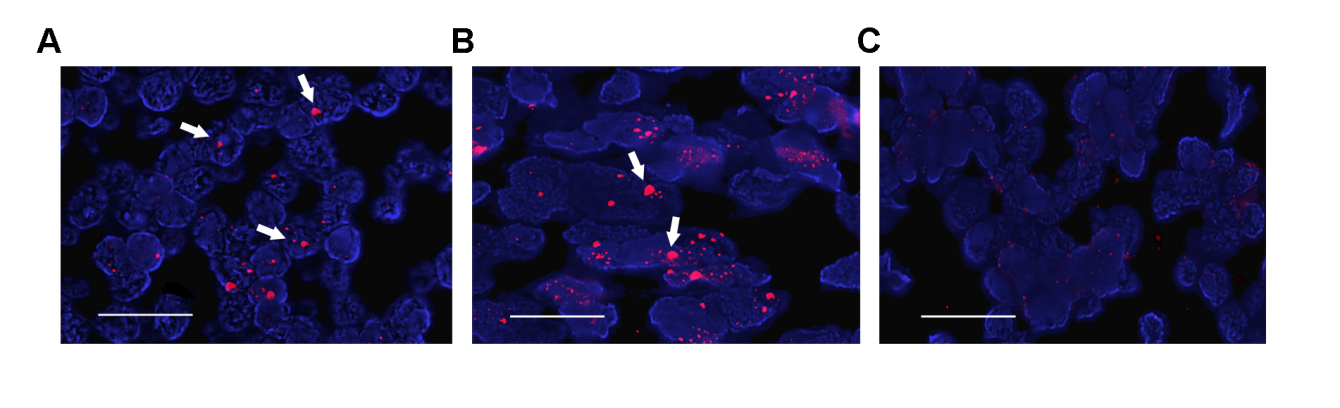


**Supplemental Figure 4. FISH performed with a PNA probe to detect telomeres.**

(**A**)Weak positive signals (red with arrows) of telomeres in HSS3T; (**B**) Strong positive signals (red with arrows) of telomeres in HSS6T; (**C**) negative signals of telomeres in HSS33T. (Scar bar: 5mm)
